# Left atrial minimum volume is more strongly associated with N-terminal pro-B-type natriuretic peptide than the left atrial maximum volume in a community-based sample

**DOI:** 10.1007/s10554-015-0800-1

**Published:** 2015-10-26

**Authors:** Pär Hedberg, Jonas Selmeryd, Jerzy Leppert, Egil Henriksen

**Affiliations:** Department of Clinical Physiology, Västmanland County Hospital, 721 89 Västerås, Sweden; Centre for Clinical Research, Västmanland County Hospital, Uppsala University, Västerås, Sweden

**Keywords:** Left atrial volumes, NT-proBNP, Doppler echocardiography, General population

## Abstract

Previous data have demonstrated that left atrial (LA) minimum volume indexed for body surface area (LAVImin) is more strongly associated with the Doppler echocardiographic E/e′ ratio than LA maximum volume index (LAVImax). We sought to explore if LAVImin was more closely related to serum levels of NT-proBNP than LAVImax and E/e′ in the community. A community-based sample of 730 subjects underwent echocardiographic examinations and NT-proBNP measurements. The mean age of the participants was 66.3 years (range 38–86) and 72 % were men. Age (Spearman correlation [rho] 0.533), LAVImin (rho 0.460), LAVImax (rho 0.360), estimated glomerular filtration rate (rho −0.349), and E/e′ (rho 0.301; all *P* < 0.001) were strongly correlated with log-NT-proBNP. In a multiple linear regression model with log-NT-proBNP as dependent variable and LAVImin, LAVImax, E/e′ ratio, and potential confounders as predictors, an adjusted R^2^ of 44.9 % was obtained. When excluding either of LAVImin (R^2^ 42.6 %, *P* < 0.001) or E/e′ (R^2^ 44.6 %, *P* = 0.019) the model fit was significantly reduced. In contrast, when LAVImax was excluded the model fit was preserved (R^2^ 45.0 %, *P* = 0.69). To detect an NT-proBNP level of >125 ng/L, LAVImin yielded a significantly larger area under the receiver operating characteristic curve (AUC) of 0.749 than LAVImax (AUC 0.701; *P* < 0.001) and E/e′ (AUC 0.661; *P* < 0.001). In our community-based sample, LAVImin was more strongly associated with NT-proBNP than LAVImax. Moreover, the discriminatory power to detect an elevated NT-proBNP level was stronger in LAVImin than in LAVImax and E/e′. Our findings support previous data that LAVImin may be more closely related to LV filling function than LAVImax.

**Electronic supplementary material:**

The online version of this article (doi:10.1007/s10554-015-0800-1) contains supplementary material, which is available to authorized users.

## Introduction

Heart failure with or without reduced left ventricular (LV) systolic function is recognized as an increasing major public health issue [1, 2]. Heart failure with preserved LV ejection fraction (LVEF), having a complex pathophysiology, is an especially challenging condition to diagnose and its diagnostic criteria are debated and still evolving [3]. A main hemodynamic abnormality in heart failure, regardless of the level of LVEF, is elevated LV filling pressure [4]. Serum levels of natriuretic peptides and Doppler echocardiography are key diagnostic methods in the clinical evaluation of patients with suspected heart failure [2]. Because natriuretic peptides are synthesized and released by cardiac myocytes primarily as a response to myocytic stretch, increased LV filling pressure causes elevation of serum natriuretic peptides [5–7]. B-type natriuretic peptide (BNP) and N-terminal pro-BNP (NT-proBNP) are well established diagnostic biological markers in heart failure and have a powerful prognostic role in various patient groups as well as in the general population [2, 8, 9]. Among commonly used Doppler echocardiographic indices, the transmitral early diastolic flow velocity/mitral annular early diastolic tissue velocity ratio (E/e′ ratio) is considered to be the most strongly associated with LV filling pressure [2, 10]. However, in patients with preserved LV systolic function, Doppler echocardiography filling indices including the E/e′ ratio has shortcomings in determining whether LV filling pressure is elevated or not [11, 12].

Left atrial (LA) maximum volume indexed for body surface area (LAVImax) has been suggested as an indicator of sustained elevation of LV filling pressure and has proved to be a robust prognostic marker in patients referred for echocardiography and in community-based populations [13, 14]. As distinct from LAVImax, LA minimum volume indexed for body surface area (LAVImin) is directly exposed to the LV end-diastolic pressure and has shown to be a better marker of the LA mechanical function than LAVImax [15, 16]. Moreover, Russo et al. [17] showed that LAVImin was more closely related to the E/e′ ratio than LAVImax in a community-based sample, suggesting that LAVImin is a more reliable marker of an increased LV filling pressure than LAVImax. To extend these findings, we sought to explore whether LAVImin is more closely related to the serum levels of NT-proBNP than LAVImax or E/e′ in a community-based sample.

## Methods

### Study population

The participants were recruited from the control group of the Västmanland Myocardial Infarction Study (VaMIS; ClinicalTrials.gov number, NCT01452178). Details of the control group recruitment have been reported elsewhere [18]. In short, consecutive patients hospitalized for acute myocardial infarction from November 2005 to May 2011 were included in the VaMIS study. For each patient included, a control subject was recruited from the general population. From the Swedish Population register, where all Swedish citizens are registered, a person with the closest date of birth, same sex, and same municipality as the VaMIS patient was identified and invited to participate by mail. All subjects underwent clinical examination, electrocardiography (ECG), echocardiographic examination, and blood sampling.

From the community-based control group of the VaMIS study (*n* = 855) we excluded individuals with non-sinus rhythm (n = 42), valvular regurgitation visually graded as more than moderate, i.e., >grade 2 of 4, or aortic valve peak systolic flow velocity of ≥4 m/s (n = 1). We further disqualified two subjects identified with premature ventricular beats in bigeminy during the echocardiographic examination and one subject in haemodialysis. Subjects with missing NT-proBNP values (n = 12), missing serum creatinine (n = 1), and non-assessable echocardiographic variables because of reduced image quality (n = 66) were excluded. Finally, 730 individuals (522 men and 208 women) were included in the analyses.

The study was approved by the Ethics Committee of Uppsala University, Sweden (Dnr 2005:382). All participants gave their written informed consent.

Self-reported diagnoses of cardiovascular disease and diabetes mellitus were confirmed from medical records. Hypertension was determined present if the participant had been assigned this diagnosis by a physician and had been prescribed antihypertensive medication. Ischemic heart disease was defined as a history of myocardial infarction, angina pectoris (confirmed by positive exercise electrocardiography, myocardial scintigraphy, or coronary angiography), coronary artery by-pass grafting, or percutaneous coronary intervention.

### Biochemistry

After participants fasted overnight, venous blood samples were taken by trained staff and immediately sent to the accredited Laboratory of Clinical Chemistry, Västmanland County Hospital, Västerås, Sweden. Plasma levels of NT-proBNP were measured by a commercially available sandwich immunoassay using monoclonal antibodies and separation based on biotin-streptavidin binding (Elecys 1010 and Cobas e411, Roche Diagnostics, Germany). The within-run coefficients of variation were 3.1 and 3.6 % for low and high levels of NT-proBNP, respectively. Glomerular filtration rate (eGFR) was estimated from serum creatinine standardized to isotope dilution mass spectrometry using the CKD-EPI equation [19].

### Echocardiographic image acquisition

A two-dimensional (2D) echocardiographic examination dedicated for research was performed using a commercially available system (Vingmed Vivid Seven, General Electric, Horten, Norway). All examinations were performed by an experienced echocardiographer (P.H.). The images were obtained in the left lateral recumbent position using a phased array transducer in the standard parasternal and apical views. The ECG-triggered 2D images and Doppler data were stored digitally in a cine loop format. Three consecutive cardiac cycles were recorded during quiet breathing.

### Echocardiographic analysis

The analysis was performed by one experienced physician (P.H.) at least 3 months after the image acquisition using commercially available software (Echo PAC, PC version 110, Horten, Norway) with anonymized images. The LV cavity and wall dimensions were measured from the 2D images. LV mass was estimated using the ASE-recommended formula [20].

LVEF was assessed by the biplane Simpson’s rule [20]. LV systolic dysfunction was defined as LVEF < 55 %. In subjects for whom it was not possible to obtain the Simpson LVEF (n = 81; 11.1 %) a visual estimation of LVEF was made.

In the assessment of LA volumes, the single-plane disc method was used in the apical 4-chamber view. The stored loops of this view were dedicated to LA visualization and oriented to maximize the LA area. LAVmax (i.e., end systolic) assessment was performed using the frame immediately preceding the mitral valve opening, and LAVmin (i.e., end diastolic) was obtained using the frame contiguous with mitral valve closure. The LA endocardial border, excluding the LA appendage and the confluences of pulmonary veins, was traced with a straight line connecting the septal and lateral mitral leaflet base attachment points to the annulus as the superior border of the outlined area.

Mitral inflow was recorded using pulsed Doppler measurements at the tips of the mitral leaflets. The peak early (E) and late (A) transmitral diastolic flow velocities were obtained. The peak velocity of the early diastolic wave (TD-e′) was measured using pulsed-wave tissue Doppler measurements with the sample volume close to the mitral valve annulus in the apical 4-chamber view in the septal (TD-e′ septal) and lateral (TD-e′ lateral) walls. The E/e′ ratio was calculated based on the transmitral E wave and the average of TD-e′ lateral and TD-e′ septal (TD-e′ mean).

The reproducibility of echocardiographic measurements was tested by having the initial examiner and another experienced physician (J.S.) at our laboratory re-measure LAVmax, LAVmin, and E/e′ from the digitally stored loops in 19 randomly selected subjects. The intra- and inter-observer reproducibility, expressed as the coefficients of variation (CVs), for the E/e′ ratio were 3.0 and 10.2 %, respectively. The corresponding CVs, as previously presented [21], for LAVmax were 8.0 and 11.9 %, respectively, and for LAVmin 16.1 and 19.2 %, respectively.

### Statistical analysis

Data are presented as mean ± standard deviation for continuous variables and as frequencies and percentages for categorical variables. The participants were categorized into three tertiles according to their serum NT-proBNP concentration. Continuous and categorical variables were compared in subgroups across tertiles using analysis of covariance and the Fisher exact test, respectively. Post-hoc analyses of differences between tertiles of NT-proBNP were presented with Bonferroni corrected *P* values. Bivariate correlations were expressed as Spearman rank order correlation coefficients (rho). Natural logarithmic transformation was applied to NT-proBNP because of its highly skewed distribution.

The independent associations between log-NT-proBNP, E/e′ ratio and LA volumes were evaluated by means of multiple general linear regression models. A full model included E/e′ ratio, LAVImax, LAVImin, and potential confounders (age, sex, smoking, body mass index, systolic and diastolic blood pressure, eGFR, diabetes, hypertension, ischemic heart disease, LV systolic dysfunction, and LV mass index). The potential confounders were chosen from previous knowledge [22] and availability in the present study. Only covariates significantly associated with NT-proBNP (Table 1) were used in the multivariable models. The model goodness-of-fit was described as the adjusted R^2^. In three separate models, either E/e′, LAVImax, or LAVImin was excluded from the full model to evaluate their discrete contribution to the association with log-NT-proBNP. The three separate nested models were compared to the full model using the likelihood ratio test. The full model was evaluated for the presence of multicollinearity by calculating the variance inflation factors for the independent variables [23]. In addition, a stepwise backward linear regression was performed to evaluate the strongest determinants of NT-proBNP using the criteria of *P* ≥ 0.05 for removal and *P* < 0.05 for re-entry into the model. The robustness of the stepwise model was assessed by 2000 bootstrap replicates.

**Table 1:** Characteristics of the study population according to tertiles of NT-proBNP levels

	All participants (n = 730)	NT-proBNP 1st tertile < 55 ng/L (n = 244)	NT-proBNP 2nd tertile 55–117 ng/L (n = 243)	NT-proBNP 3rd tertile > 117 ng/L (n = 243)	*P* value overall
Male sex	522 (72 %)	212 (87 %)	173 (71 %)***	137 (56 %)***^,††^	<0.001
Age (years)	66 ± 9	60 ± 9	66 ± 8***	72 ± 7***^,†††^	<0.001
Smoking	76 (10 %)	30 (12 %)	28 (12 %)	18 (7 %)	0.16
Height (cm)	173 ± 9	176 ± 8	173 ± 9**	170 ± 10***^,†††^	<0.001
Weight (kg)	80 ± 14	84 ± 13	79 ± 13***	76 ± 15***^,†^	<0.001
Body mass index (kg/m^2^)	26.5 ± 3.6	27.0 ± 3.4	26.3 ± 3.5	26.2 ± 4.0	0.047
Body surface area (m^2^)	1.96 ± 0.21	2.03 ± 0.19	1.95 ± 0.20***	1.89 ± 0.22***^,††^	<0.001
Hypertension	259 (35 %)	49 (20 %)	86 (35 %)**	124 (51 %)***^,††^	<0.001
Diabetes mellitus	62 (8 %)	16 (7 %)	22 (9 %)	24 (10 %)	0.38
Ischemic heart disease	57 (8 %)	8 (3 %)	13 (5 %)	36 (15 %)***^,††^	<0.001
Systolic BP (mmHg)	149 ± 20	146 ± 19	147 ± 19	155 ± 22***^,†††^	<0.001
Diastolic BP (mmHg)	83 ± 10	84 ± 10	83 ± 10	82 ± 10*	0.028
eGFR (mL/min per 1.73 m^2^)	81 ± 15	86 ± 13	82 ± 15**	75 ± 15***^,†††^	<0.001
*Echocardiography*					
IVSd (mm)	11 ± 2	11 ± 2	11 ± 2	11 ± 2	0.47
LVPWd (mm)	10 ± 2	10 ± 1	10 ± 1	10 ± 2	0.51
RWT	0.43 ± 0.08	0.43 ± 0.06	0.43 ± 0.07	0.44 ± 0.09	0.33
LV mass index (g/m^2^)	99 ± 22	95 ± 19	97 ± 19	104 ± 27***^,††^	<0.001
LV systolic dysfunction	37 (5 %)	4 (2 %)	10 (4 %)	23 (10 %)**	<0.001
Mitral flow E wave (cm/s)	57 ± 14	56 ± 11	56 ± 14	59 ± 16*^,†^	0.009
Mitral flow A wave (cm/s)	58 ± 16	54 ± 13	59 ± 15**	62 ± 18***	<0.001
E/A ratio	1.03 ± 0.35	1.08 ± .34	0.99 ± 0.30*	1.03 ± .40	0.016
E/e′ ratio	7.5 ± 2.3	6.8 ± 1.7	7.3 ± 1.9*	8.5 ± 2.8***^,†††^	<0.001
LAVImin (mL/m^2^)	14 ± 7	11 ± 4	13 ± 5**	18 ± 8***^,†††^	<0.001
LAVImax (mL/m^2^)	29 ± 9	25 ± 7	27 ± 7*	33 ± 11***^,†††^	<0.001

Values are mean ± SD or number (percentages)

*BP* blood pressure, *IVSd* diastolic thickness of interventricular septum thickness, *LAVImax* left atrial maximum volume indexed for body surface area, *LAVImin* left atrial minimum volume indexed for body surface area, *LV* left ventricle, *LVPWd* diastolic thickness of left ventricular posterior wall, *RWT* relative wall thickness

* *P* < 0.05 versus 1st tertile; ** *P* < 0.01 versus 1st tertile; *** *P* < 0.001 versus 1st tertile; ^†^ *P* < 0.05 versus 2nd tertile; ^††^ *P* < 0.01 versus 2nd tertile; ^†††^ *P* < 0.001 versus 2nd tertile

In sensitivity analyses we performed the multiple linear regression comparisons by including LVEF as a continuous variable rather than as dichotomized, by excluding subjects with LVEF < 55 %, or by including LA volumes indexed to allometric height^2.7^ [24, 25] rather than to body surface area.

The discriminatory power of E/e′, LAVImax, and LAVImin to detect an increased NT-proBNP level was evaluated by the areas under the receiver operating characteristic curves (AUC). A cut-off value of 125 ng/L was chosen for NT-proBNP as suggested by the European Society of Cardiology guidelines in evaluating non-acute heart failure [2]. In post hoc testing between AUCs, Bonferroni correction of *P* values was applied.

Finally, differences in characteristics between the subjects excluded because of missing data and the participants were compared using the Fisher exact test for categorical variables and an unpaired *t* test for continuous variables. To identify characteristics independently associated with missing data, variables significant in the univariate analysis were included as covariates in a logistic regression model with missingness of data (present/absent) as dependent variable. STATA version 14 (StataCorp LP, College Station, TX, USA) was used for all analyses. *P* < 0.05 was considered significant.

## Results

The mean age of the 730 participants in the present analysis was 66.3 ± 9.5 years (range 38–86) and 72 % (n = 522) were men. The distribution of NT-proBNP levels was positively skewed with the median at 78 ng/L (interquartile range 42–154) and the 95th percentile at 504 ng/L. Thirty-two percent (n = 232) of the participants had NT-proBNP level of >125 ng/L. Table 1 presents the characteristics of the participants according to tertiles of NT-proBNP level. Compared with those having the lowest NT-proBNP levels, the subjects with the highest NT-proBNP levels were older and more likely to be women. Higher NT-proBNP levels were also related to higher prevalence of hypertension and ischaemic heart disease, to higher systolic blood pressure, and lower diastolic blood pressure. Among echocardiographic variables, LV mass index, LV systolic dysfunction, mitral inflow A wave, E/e′, and left atrial volumes were the most strongly associated with higher NT-proBNP levels.

**Fig. 1:**
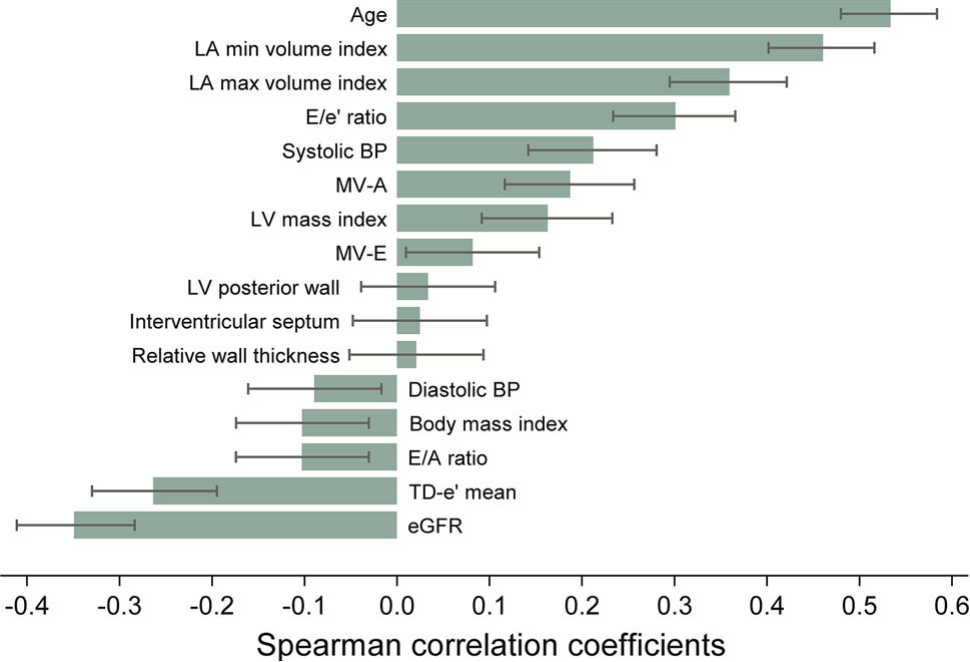
Bivariate Spearman rank order correlations with log-NT-proBNP. *Main bars* are correlation coefficients and *error bars* are 95 % confidence intervals. *BP* blood pressure; eGFR estimated glomerular filtration rate; *LA* left atrium; *LV* left ventricle; *MV*-*A* mitral valve flow A wave; *MV*-*E* mitral valve flow E wave; *TD*-*e′ mean* tissue Doppler e′ wave average of septal and lateral wall

Age (rho 0.533), LAVImin (rho 0.460), LAVImax (rho 0.360), eGFR (rho −0.349), and E/e′ (rho 0.301; all *P* < 0.001) were among the variables with the largest bivariate correlation coefficients with log-NT-proBNP (Fig. 1). The correlation between LAVImin and LAVImax was 0.765 (*P* < 0.001). The correlation coefficients for LAVImin and LAVImax with E/e′ were 0.147 (*P* < 0.001) and 0.078 (*P* = 0.034), respectively.

In a multiple linear regression model with log-NT-proBNP as dependent variable and LAVImin, LAVImax, E/e′, and potential confounders as predictors, an adjusted R^2^ of 44.9 % was obtained (Table 2). When excluding LAVImax, the regression model goodness-of-fit was preserved with an adjusted R^2^ of 45.0 % (χ^2^ 0.16, *P* = 0.69). However, when either E/e′ or LAVImin was excluded, the model fit was reduced to adjusted R^2^ of 44.6 % (χ^2^ 5.53, *P* = 0.019) and 42.6 % (χ^2^ 31.68, *P* < 0.001), respectively. All variance inflation factors for the independent variables were below 4. Sensitivity analyses did not appreciably alter the results (see Online Resource).

**Table 2:** Multiple linear regression analyses with log-NT-proBNP as the dependent variable

	β	*P*	Adjusted R^2^ (%)	Chi^2†^	*P* ^†^
Full model^a^			44.9	–	–
E/e′ ratio (+1 SD)	0.086	0.020			
LAVImax (+1 SD)	−0.020	0.70			
LAVImin (+1 SD)	0.307	<0.001			
Full model^a^ excluding E/e′ ratio			44.6	5.53	0.019
E/e′ ratio (+1 SD)	–	–			
LAVImax (+1 SD)	−0.029	0.57			
LAVImin (+1 SD)	0.336	<0.001			
Full model^a^ excluding LAVImax			45.0	0.16	0.69
E/e′ ratio (+1 SD)	0.087	0.018			
LAVImax (+1 SD)	–	–			
LAVImin (+1 SD)	0.291	<0.001			
Full model^a^ excluding LAVImin			42.6	31.68	<0.001
E/e′ ratio (+1 SD)	0.134	<0.001			
LAVImax (+1 SD)	0.196	<0.001			
LAVImin (+1 SD)	–	–			

^a^Full model included E/e′ ratio, LAVImax, LAVImin, age, sex, smoking, body mass index, systolic BP, diastolic BP, estimated glomerular filtration rate, diabetes, hypertension, ischaemic heart disease, LV systolic dysfunction, LV mass index, and mitral valve flow A-wave

^†^Likelihood ratio test Chi^2^ and *P* value in comparison with full model

In a stepwise backward linear regression analysis, age, sex, and LAVImin were strong determinants of log-NT-proBNP (Table 3). Moreover, these three predictors were the only ones to be retained in >99 % of the 2000 bootstrap replications of the stepwise regression model. LAVImax and E/e′ ratio were retained in 7.1 and 60.9 %, respectively, of the bootstrap replicates.

**Table 3:** Determinants of log-NT-proBNP in stepwise backward linear regression

	β*	*P* value*	BIF^a^ (%)
Age (+1 SD)	0.273	<0.001	100.0
Male sex (yes)	−0.400	<0.001	99.9
Smoking (yes)	–	–	10.2
Body mass index (+1 SD)	−0.079	0.009	71.7
Systolic BP (+1 SD)	–	–	42.8
Diastolic BP (+1 SD)	–	–	10.6
eGFR (+1 SD)	−0.070	0.036	53.7
Diabetes (yes)	–	–	9.0
Hypertension (yes)	0.210	0.001	79.4
Ischaemic heart disease (yes)	–	–	56.6
LV systolic dysfunction (yes)	0.394	0.004	78.0
LV mass index (+1 SD)	0.095	0.004	67.4
Mitral valve flow A wave (+1 SD)	−0.078	0.032	57.4
E/e′ ratio (+1 SD)	0.090	0.015	60.9
LAVImax (+1 SD)	–	–	7.1
LAVImin (+1 SD)	0.297	<0.001	100.0

* Regression coefficients and *P* values for the selected variables in a stepwise backward linear regression. All variables in the table were included at start

^a^Bootstrap inclusion fraction, i.e., the fraction of occasions that a variable was selected by stepwise backward linear regression in 2000 bootstrapped sample replicates

**Fig. 2:**
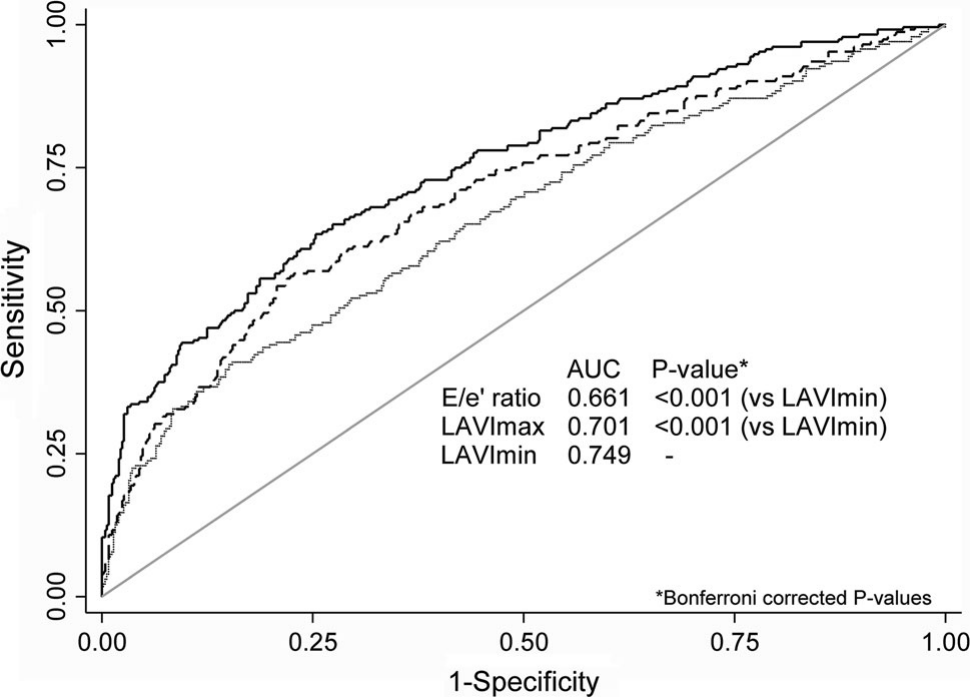
Receiver operating characteristic curves for left atrial minimum volume index (LAVImin; *solid line*), left atrial maximum volume index (LAVImax; *dashed line*), and E/e′ ratio (*dotted line*) in detecting NT-proBNP level of >125 ng/L. Among the 730 participants 32 % (n = 232) had an NT-proBNP level of >125 ng/L

Figure 2 shows the ability of LAVImin, LAVImax, and E/e′ ratio to detect an NT-proBNP level of >125 ng/L. LAVImin yielded a significantly higher AUC of 0.749 than LAVImax (AUC 0.701; *P* < 0.001) and E/e′ ratio (AUC 0.661; *P* < 0.001).

Subjects excluded because of missing data (n = 79) were more often women (44 vs. 28 %; *P* = 0.006), had more often hypertension (48 vs. 36 %; *P* = 0.036), and greater body mass index (28.1 ± 4.7 vs. 26.5 ± 3.6; *P* < 0.001) compared with the participants. There were no significant differences between excluded subjects and participants regarding age, smoking, diabetes, ischaemic heart disease, and systolic and diastolic blood pressure. In a logistic regression model including sex, hypertension, and body mass index as covariates, sex (*P* = 0.006) and body mass index (*P* = 0.004) remained as independent covariates associated with missing data.

## Discussion

In the present community-based, LAVImin was a significantly stronger determinant of NT-proBNP than LAVImax after adjustment for potential confounders. Further, LAVImin yielded a significantly larger AUC than LAVImax and E/e′ to detect NT-proBNP above a threshold level of 125 ng/L.

Investigators have struggled to define and to agree upon which non-invasive parameters should be used to identify subjects with increased LV filling pressure [26–28]. The traditional classification system based on transmitral and tissue Doppler indices has been well validated in patients with heart failure with reduced LVEF, but is known to have shortcomings in patients with decompensated heart failure with preserved LVEF [11, 12].

In contrast with Doppler filling indices, which are influenced by short-term changes in filling pressure, the LA volume has been proposed as an indicator of long-lasting diastolic burden with a slow and persistent remodelling reflecting a chronic pressure or volume load [13, 29]. However, it has been shown that the LA reservoir volume (LAVImax) depends not only on the present LA pressure and relaxation, but is also reliant on the LV systolic mitral annular excursion [17]. Because the LA reservoir volume is influenced by the LV contractile state, the LV systole may act as a confounder in the relationship between LV diastolic function and LAVImax [17]. In contrast with the LA reservoir volume, the LA is directly exposed to the LV pressure during the conduit and atrial contraction phases. An increase in LV filling pressure may therefore directly affect LA wall tension during diastasis and atrial contraction and, therefore, may lead to an increase of LAVImin.

Increased LV filling pressure causes an elevation of serum BNP and NT-proBNP in response to cardiac myocytic stretch [6, 7]. Several studies have confirmed the positive correlation between LAVImax and BNP and LAVImax is recognized as an independent predictor of NT-proBNP [30, 31]. Russo et al. [17] showed that the E/e′ ratio better correlated with LAVImin than with LAVImax proposing LAVImin to be better predictor of LV filling disorders than LAVImax. If true, one could assume an elevated NT-proBNP to be more closely related to LAVImin than to LAVImax. We confirmed the finding by Russo et al. that LAVImin is more closely related to E/e′ than LAVImax. For outpatients presenting with non-acute symptoms of possible heart failure the European Society of Cardiology guidelines recommends an NT-proBNP serum level of 125 ng/L as an optimal cut-off point for excluding heart failure [2]. Using this cut-off as a marker of possible heart failure, we showed that LAVImin had a significantly stronger discriminatory ability to detect an elevated NT-proBNP than LAVImax and E/e′. Thus, the present data supports recent reports showing LAVImin to be more closely related to LV filling than LAVImax.

We previously demonstrated that LAVImax was independent of age and sex in a healthy population-based sample, whereas LAVImin significantly increased with age [21]. Further, the intra- and inter-observer reproducibility of LA volume measurements has been shown to be slightly poorer for LAVImin compared with LAVImax [21, 32]. LAVImin may be more challenging to manage in a clinical setting than LAVImax because of its age dependency and its less beneficial reproducibility.

Several investigators have demonstrated the utility of LAVImax in predicting cardiovascular outcome in unselected community-based cohorts whereas data are limited for LAVImin [14, 33, 34]. In 178 consecutive outpatients, Caselli et al. [16] found that LAVImin, as measured by 3-dimensional echocardiography, was the most powerful echocardiographic predictor of adverse cardiovascular events. Abhayaratna et al. [15] found that a reduced LA emptying fraction, mainly driven by an increased LAVImin, was a more powerful predictor of the incidence of atrial fibrillation than the LAVImax. Because NT-proBNP is a significant independent predictor of all-cause mortality and cardiovascular events [8] one may hypothesize LAVImin to be a stronger prognostic predictor than LAVImax, but this remains to be determined.

## Limitations

Our conclusions are limited to community-based adult Caucasian subjects. Further, the female portion of the study population was only 28 %. The reason for this under-representation of women was that, for each patient included in the VaMIS study, a control subject matched for age and sex was recruited from the general population. Consequently, the sex distribution of the control subjects who were enrolled in the present study reflects the sex difference in patients hospitalized for myocardial infarction. Nearly 10 % of the eligible subjects were excluded due to missing values, mainly because of reduced echocardiographic image quality. With the exception of sex and body mass index, there were no differences in characteristics between the excluded subjects and the participants. However, it cannot be excluded that selection bias could partially influence our results.

The acquisition and storage of cine loops dedicated to LA planimetry were only obtained in the 4-chamber view, and not in the 2-chamber view. Thus, the guideline-recommended biplane assessment of LA volumes [20] was not possible, which is a limitation of the study. Given the association of body mass index with NT-proBNP levels, the guideline recommended method of indexing the LA volumes to body surface area [20] may have affected our results. However, sensitivity analyses using LA volumes indexed to allometric height^2.7^ did not appreciably change our conclusions.

It must be clarified that NT-proBNP, E/e**′**, and LA volumes are just surrogate markers for LV filling pressure and that no invasive pressure measurements were made in the present study. Grading of LV diastolic function may have been of interest in our analyses. However, due to the lack of consensus on appropriate variables and algorithms to define diastolic function, as recently demonstrated by our group [35], we chose to avoid analysing diastolic dysfunction beyond LV filling pressure.

Multicollinearity may be a problem in multiple regression models when including strongly correlated variables, such as LAVImin and LAVImax, in the same model. However, the variance inflation factors for the independent variables were all <4 as an indicator of a low degree of collinearity in the present study. Moreover, in multiple linear regression modelling, collinearity affects regression coefficients and standard errors of the independent variables but it does not have an impact of the overall model fit [36]. The present study design was limited to cross-sectional associations between the studied variables; thus, further work is required to evaluate the prognostic impact of LAVImin.

## Conclusions

In the present population-based sample, LAVImin was more strongly associated with NT-proBNP than LAVImax. Moreover, the discriminatory power to detect an NT-proBNP level of >125 ng/L was stronger in LAVImin than in LAVImax and E/e′. Our findings support and extend previous data suggesting that LAVImin may be more closely related to LV filling function than LAVImax in a community-based population. Future studies are warranted to evaluate the potential role of LAVImin as a sensitive screening tool for LV filling dysfunction.

## Electronic supplementary material

Supplementary material 1 (PDF 243 kb)
