# Supplementary material for: Left atrial minimum volume is more strongly associated with N-terminal pro-B-type natriuretic peptide than the left atrial maximum volume in a community-based sample
Source: Int J Cardiovasc Imaging. 2015 Oct 26;32:417–25. doi: 10.1007/s10554-015-0800-1 (PMC4751167; doi:10.1007/s10554-015-0800-1)
Supplement: Supplementary file 1 — Supplementary material 1 (PDF 243 kb) [file 10554_2015_800_MOESM1_ESM.pdf]

Supplementary material

Left atrial minimum volume is more strongly associated with N-terminal pro-B-type natriuretic peptide than the left atrial maximum volume in a community-based sample

International Journal of Cardiovascular Imaging

Pär Hedberg, Jonas Selmeryd, Jerzy Leppert, Egil Henriksen

Correspondence:
Dr Pär Hedberg
Department of Clinical Physiology, Västmanland County Hospital,
SE-72 189 Västerås, Sweden.
E-mail: par.o.hedberg@ltv.se

Supplementary Table 1 Multiple linear regression analyses with log-NT-proBNP as the dependent variable. Left ventricular ejection fraction (LVEF) was entered in the model as a continuous variable rather than as dichotomized. Consequently, subjects with non-assessable Simpson LVEF were excluded, leaving 649 participants to be analysed.

	**β**	***P***	**Adjusted R^2**	**$Chi^{2\dagger}$**	***P*†**
**Full model***			46.2%	-	-
E/e′ ratio (+1 SD)	0.077	0.049			
LAVImax (+1 SD)	-0.023	0.68			
LAVImin (+1 SD)	0.320	<0.001			
**Full model* excluding E/e′ ratio**			45.9%	3.98	0.046
E/e′ ratio (+1 SD)	-	-			
LAVImax (+1 SD)	-0.033	0.54			
LAVImin (+1 SD)	0.346	<0.001			
**Full model* excluding LAVImax**			46.2%	0.18	0.67
E/e′ ratio (+1 SD)	0.079	0.044			
LAVImax (+1 SD)	-	-			
LAVImin (+1 SD)	0.301	<0.001			
**Full model* excluding LAVImin**			43.8%	29.39	<0.001
E/e′ ratio (+1 SD)	0.125	0.001			
LAVImax (+1 SD)	0.201	<0.001			
LAVImin (+1 SD)	-	-			

*Full model included E/e′ ratio, LAVImax, LAVImin, age, sex, smoking, body mass index, systolic BP, diastolic BP, estimated glomerular filtration rate, diabetes, hypertension, ischaemic heart disease, LV ejection fraction, LV mass index, and mitral valve flow A-wave.
†Likelihood ratio test Chi^2 and *P*-value in comparison with full model.

Supplementary Table 2 Multiple linear regression analyses with log-NT-proBNP as the dependent variable. Subjects with left ventricular ejection fraction <55 % were excluded leaving 693 participants to be analysed.

	**β**	***P***	**Adjusted R^2**	**Chi^{2†}**	***P*†**
**Full model***			41.0%	-	-
E/e′ ratio (+1 SD)	0.065	0.098			
LAVImax (+1 SD)	0.029	0.58			
LAVImin (+1 SD)	0.244	<0.001			
**Full model* excluding E/e′ ratio**			40.8%	2.80	0.094
E/e′ ratio (+1 SD)	-	-			
LAVImax (+1 SD)	0.028	0.60			
LAVImin (+1 SD)	0.257	<0.001			
**Full model* excluding LAVImax**			41.0%	0.32	0.57
E/e′ ratio (+1 SD)	0.064	0.10			
LAVImax (+1 SD)	-	-			
LAVImin (+1 SD)	0.269	<0.001			
**Full model* excluding LAVImin**			39.5%	17.66	<0.001
E/e′ ratio (+1 SD)	0.087	0.028			
LAVImax (+1 SD)	0.197	<0.001			
LAVImin (+1 SD)	-	-			

*Full model included E/e′ ratio, LAVImax, LAVImin, age, sex, smoking, body mass index, systolic BP, diastolic BP, estimated glomerular filtration rate, diabetes, hypertension, ischaemic heart disease, LV mass index, and mitral valve flow A-wave.

†Likelihood ratio test Chi^2 and *P*-value in comparison with full model.

Supplementary Table 3 Multiple linear regression analyses with log-NT-proBNP as the dependent variable (n = 730). Left atrial minimum and maximum volumes were indexed to allometric height$^{2.7}$ rather than to body surface area.

	**β**	***P***	**Adjusted R^2**	**$Chi^{2\dagger}$**	***P*†**
**Full model***			44.7%	-	-
E/e′ ratio (+1 SD)	0.086	0.021			
LAVImax (+1 SD)	-0.030	0.58			
LAVImin (+1 SD)	0.318	<0.001			
**Full model* excluding E/e′ ratio**			44.4%	5.44	0.020
E/e′ ratio (+1 SD)	-	-			
LAVImax (+1 SD)	-0.039	0.46			
LAVImin (+1 SD)	0.348	<0.001			
**Full model* excluding LAVImax**			44.8%	0.32	0.57
E/e′ ratio (+1 SD)	0.087	0.019			
LAVImax (+1 SD)	-	-			
LAVImin (+1 SD)	0.293	<0.001			
**Full model* excluding LAVImin**			42.3%	31.49	<0.001
E/e′ ratio (+1 SD)	0.134	<0.001			
LAVImax (+1 SD)	0.197	<0.001			
LAVImin (+1 SD)	-	-			

*Full model included E/e′ ratio, LAVImax, LAVImin, age, sex, smoking, body mass index, systolic BP, diastolic BP, estimated glomerular filtration rate, diabetes, hypertension, ischaemic heart disease, LV systolic dysfunction, LV mass index, and mitral valve flow A-wave.
†Likelihood ratio test Chi^2 and *P*-value in comparison with full model.
